# How Glutamate Is Managed by the Blood–Brain Barrier

**DOI:** 10.3390/biology5040037

**Published:** 2016-10-08

**Authors:** Richard A. Hawkins, Juan R. Viña

**Affiliations:** 1 Department of Physiology and Biophysics, The Chicago Medical School, Rosalind Franklin University of Medicine and Science, North Chicago, IL 60088, USA; 2 Department of Biochemistry and Molecular Biology, Faculty of Medicine and Odontology, Medical Research Institute-INCLIVA, University of Valencia, Valencia 46010, Spain; Juan.R.Vina@uv.es

**Keywords:** glutamate, glutamine, BBB (blood–brain barrier), brain, oxoproline, amino acid transport

## Abstract

A facilitative transport system exists on the blood–brain barrier (BBB) that has been tacitly assumed to be a path for glutamate entry to the brain. However, glutamate is a non-essential amino acid whose brain content is much greater than plasma, and studies in vivo show that glutamate does not enter the brain in appreciable quantities except in those small regions with fenestrated capillaries (circumventricular organs). The situation became understandable when luminal (blood facing) and abluminal (brain facing) membranes were isolated and studied separately. Facilitative transport of glutamate and glutamine exists only on the luminal membranes, whereas Na^+^-dependent transport systems for glutamate, glutamine, and some other amino acids are present only on the abluminal membrane. The Na^+^-dependent cotransporters of the abluminal membrane are in a position to actively transport amino acids from the extracellular fluid (ECF) into the endothelial cells of the BBB. These powerful secondary active transporters couple with the energy of the Na^+^-gradient to move glutamate and glutamine into endothelial cells, whereupon glutamate can exit to the blood on the luminal facilitative glutamate transporter. Glutamine may also exit the brain via separate facilitative transport system that exists on the luminal membranes, or glutamine can be hydrolyzed to glutamate within the BBB, thereby releasing ammonia that is freely diffusible. The γ-glutamyl cycle participates indirectly by producing oxoproline (pyroglutamate), which stimulates almost all secondary active transporters yet discovered in the abluminal membranes of the BBB.

## 1. Introduction

The blood–brain barrier (BBB) envelops the whole central nervous system (CNS). The endothelial cells of cerebral capillaries impede the free movement of hydrophilic molecules into the brain. Furthermore, it is the luminal (blood-facing) and abluminal (brain-facing) membranes of the endothelial cells that provide primary resistance to the movement of molecules [1].

## 2. Glutamate in the Brain and the Circulation

Glutamate is a non-essential amino acid. However, it is the most abundant free amino acid in the brain. Glutamate concentrations in plasma are 50–100 µM; in the whole brain, they are 12 µmol/g, but only 0.5–2 µM in extracellular fluids (ECF).

## 3. Glutamate in ECF Cannot Be Permitted to Increase

Large doses of glutamate, given by injection, caused damage to the brain in areas that were not protected by the BBB [1,2,3,4,5]. Subsequently, the hypothesis emerged that neuronal death could be produced by overstimulation of excitatory amino acid receptors; this became a popular explanation of the pathogenesis of neuronal death. Ischemic episodes can lead to the release of glutamate [6,7] from brain cells and an excessive accumulation of glutamate in the ECF [8,9]. The overexcitation of neurons by glutamate may result in a calcium influx that activates catabolic enzymes, resulting in neuronal death [10].

## 4. Facilitative and Active Transport Systems for Glutamate in the BBB

Studies in vivo found facilitative transporters in the BBB that are saturable and stereoselective [11,12,13]. It was deduced that these transporters are present in the luminal membrane. On the other hand, it has been shown that glutamate does not enter the brain in appreciable quantities, except in the circumventricular organs [14,15,16]. This was puzzling and raised a question: why should there be a facilitative transport system for glutamate when glutamate is synthesized by the brain in large quantities? Studies of both sides of the BBB separately were necessary to answer the question.

## 5. Studying Each Side of the BBB Separately

**Figure 1:**
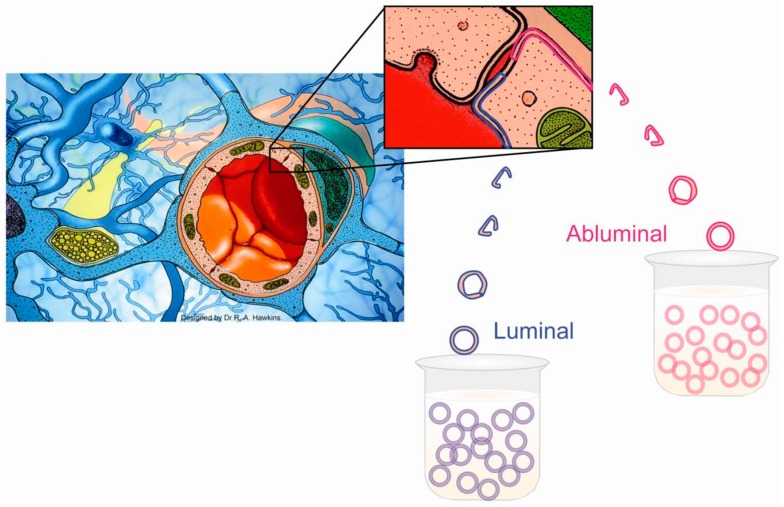
Diagrammatic representation of the blood–brain barrier and isolated luminal and abluminal membranes. The BBB exists at the level of the endothelial cells of cerebral capillaries. The endothelial cells are joined together by an extensive network of tight junctions. A basement membrane, within which pericytes reside, surrounds the endothelial cells, as does a layer comprised of astrocyte processes (so called end-feet). The pericytes are numerous and most likely function as phagocytes. The astrocyte layer serves as a metabolic barrier. For instance, astrocytes incorporate NH_4_^+^ into glutamine, and metabolize short-chain fatty acids. Capillaries are collected from the bovine cerebral cortex, and their membranes detached [17,18]. The luminal and abluminal membranes are isolated by differential centrifugation. The membranes form sealed spheres that are suitable for the study of transport. It is possible, for instance, to create trans-membrane potentials, and establish external Na^+^ gradients testing for the presence of Na^+^-dependent transport systems. This illustration is modified from a figure in [19].

Studies of the BBB in vivo gave incomplete information because they did not take into account the fact that metabolites have to pass through both the luminal and abluminal membranes to gain access to brain cells. To provide a suitable in vitro model, plasma membranes of the endothelial cells were separated and found to be suitable for the study of transport in vitro [17]. With isolated membranes it was possible to demonstrate differences between the two sides of the BBB (polarity), showing that facilitative carriers for glutamate exist exclusively in the luminal membranes and energy-dependent Na^+^-co-transporters are present only in the abluminal membrane (Figure 1). Recently, it has been shown using isolated brain capillaries that the amount of proteins from different transporters is very similar between the marmoset and humans, but there are significant differences when compared to rats [18].

## 6. Facilitative Transport of Glutamate Is Restricted to Luminal Membranes

Lee et al. [20] found, as expected, a facilitative glutamate transport on the luminal membrane, but glutamate transport activity on the abluminal membrane was not detectable. Therefore, while glutamate may enter endothelial cells, it can go no further; no transport of glutamate is possible from the endothelial cells into the brain.

Several groups have found that luminal membrane carriers of amino acids have no dependence on Na^+^ gradients [21,22,23,24,25,26] and are therefore energy-independent. As mentioned, the presence of a transporter for acidic amino acids that had a high affinity and a low capacity [4,11,25,26] was an enigma for many years because both glutamate and aspartate are non-essential amino acids that are synthesized and accumulated in high concentrations in the brain.

## 7. Active Transport Systems of the BBB Can Expel Glutamate from the ECF

ECF glutamate concentration is low (≈ 0.5–2 µM) [4]. The gradient between brain cells and ECF is maintained by a family of Na^+^-dependent glutamate transporters known as excitatory amino acid transporters (EAAT). These transporters couple with the steep Na^+^ gradient that normally exists between the ECF and brain cells. Currently, five members of the EAAT family have been identified. They reside in the plasma membranes of astrocytes and neurons [27,28,29,30,31] as well as the BBB [32].Three EAAT members are found in the abluminal membrane of the BBB, in a position to maintain the glutamate gradient between the brain cells and ECF.

**Figure 2:**
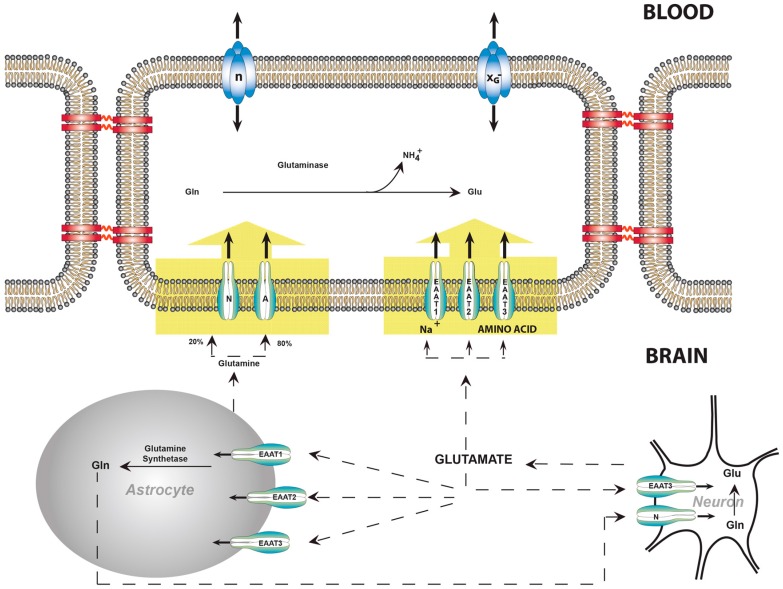
Glutamate and glutamine transport between neurons, astrocytes, and endothelial cells of the blood–brain barrier. Glutamate is the most abundant excitatory neurotransmitter in the mammalian nervous system. At chemical synapses, glutamate is stored in vesicles. Nerve impulses trigger the release of glutamate from the pre-synaptic cell. Na^+^-dependent glutamate transporters (EAATs) are found in neuronal and glial membranes [27,28,29,30,31]. These transporters play the important role of regulating concentrations of glutamate in the extracellular space, keeping it at low levels. After glutamate is released as the result of an action potential, glutamate transporters quickly remove it from the extracellular space, thereby terminating the synaptic transmission. Without the activity of glutamate transporters, glutamate would accumulate and kill cells in a process called excitotoxicity, in which excessive amounts of glutamate act as a toxin to neurons [4]. The activity of these transporters also allows glutamate to be recycled. In brain injury or oxygen insufficiency, the EAATs can work in reverse and excess glutamate can accumulate outside cells, rapidly halting neurotransmission. At least three EAATs are present in the abluminal membrane of the BBB [32]. These EAATs move glutamate into the endothelial cell, from which egress is possible through the facilitative transporters in the luminal membrane. There are transporters capable of pumping glutamine from ECF into endothelial cells; glutaminase within endothelial cells may also hydrolyze glutamine to glutamate and NH_4_^+^. No carrier is necessary for NH_4_^+^, which may diffuse as NH_3_^+^. Abbreviations: A, Na^+^-dependent system A; N, Na^+^-dependent system N; EAAT, Na^+^-dependent glutamate transporter, the lightning symbols indicate Na^+^-dependence, X_G_^−^, facilitative amino acid transporter for glutamate. This illustration is modified from a figure in [33].

The cDNAs for EAAT 1, 2, and 3 were isolated from the endothelial cells of cerebral capillaries [32]. Western blot analysis established that these glutamate transporters are present exclusively in the abluminal membranes (Figure 2); no EAATs were detectable in luminal membranes [32,34]. Other EAATs were not investigated, although a transcript for EAAT4 was detected in isolated endothelial cells [30]. EAATs 1, 2, and 3 were demonstrated to be voltage-dependent, and collectively have an apparent Km of 14 µM at a transmembrane potential of −61mV [32]. The EAAT family is the most forceful of the Na^+^-dependent amino acid transporters found in the abluminal membranes to date [35,36].

## 8. Balance of Glutamate, Glutamine, and Ammonia

While most ammonia that passes through the BBB is incorporated into the amide group of glutamine by astrocytes [37,38,39], it has not been possible to consistently measure arteriovenous differences in NH_4_^+^. Furthermore, if there were no mechanism for the removal of glutamine it would accumulate in the brain, thereby raising the osmolarity and causing swelling. The situation is now explicable; glutamine and glutamate are pumped from ECF into endothelial cells by Na^+^-dependent transport systems [20,32]. Glutamine is at least partially metabolized to NH_4_^+^ and glutamate within endothelial cells. The remaining glutamine as well as NH_4_^+^ and glutamate are free to diffuse across the luminal membrane into the blood [20]. In this way the NH_4_^+^ uptake and release may be balanced. Therefore, the BBB participates in the regulation of brain nitrogen metabolism, preventing the accumulation of glutamate and glutamine as well as NH_4_^+^ (Figure 2).

## 9. Transport of Amino Acids across the BBB and the Role of Oxoproline

Many tissues that actively transport amino acids have high γ-glutamyltranspeptidasea ctivity [40,41]. These include: the brush border of the proximal convoluted tubules of the kidney [42], lactating mammary glands [43], the apical portion of the intestinal epithelium [44], the choroid plexus [45], and the BBB [46,47,48].

**Figure 3:**
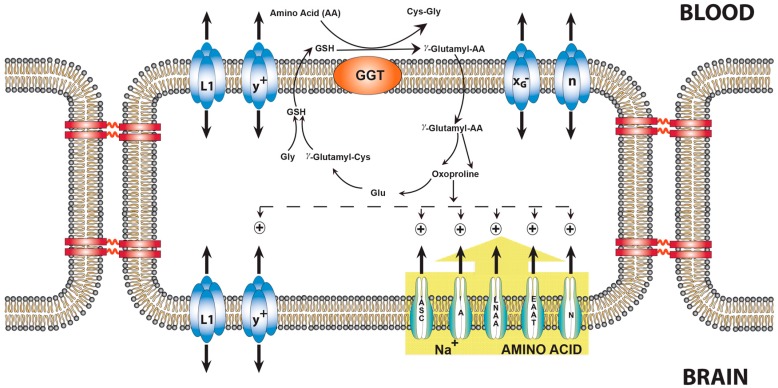
The influence of oxoproline on AA transport across the blood–brain barrier. γ-Glutamyl-AAs are formed at the outer surface of the luminal membranes of the endothelial cells that transfer the γ-glutamyl moiety of glutathione to most AA, thereby forming a γ-glutamyl-AA. The γ-glutamyl-AA enters endothelial cells, where the AA is released and oxoproline is formed. The Na^+^-dependent transport systems A, ASC, Na^+^-LNAA, EAAT, and y^+^, all located on the abluminal side, are activated by oxoproline [49]. System N was the only system not stimulated. L1 is present on both the luminal and abluminal membrane and is not affected by oxoproline [47]. Abbreviations: A, Na^+^-dependent system A; N, Na^+^-dependent system N; EAAT, Na^+^-dependent glutamate transporter, X_G_^−^, facilitative glutamate transporter, n, facilitative glutamine transporter. The possibility exists that oxoproline causes an increase in the transmembrane potential, therefore providing a greater driving force. All transport systems indicated by a + above them are stimulated by oxoproline.This illustration is modified from a figure in [19].

Using the placenta and the lactating mammary gland as experimental models, it was shown that oxoproline, an intermediate of the γ-glutamyl cycle, serves as an intracellular signal to stimulate Na+-dependent amino acid uptake [43]. Although it has been suggested that the main role of gamma-glutamyltransferase is as a hydrolase rather than a transpeptidase, there is evidence for the natural occurrence of gamma-glutamyl compounds. Oxoproline stimulates several Na^+^-dependent amino acid transporters located in the abluminal membranes of the BBB, including those that transport glutamate and glutamine [49] (Figure 3).

## 10. Conclusions

The current concept of the BBB is that cerebral endothelial cells are not passive barriers; rather, they participate actively in regulating the composition of brain ECF. The two membranes seem to be working in a complementary fashion with, for the most part, active transport occurring at the abluminal membrane and facilitative transport at the luminal membrane. The abluminal membrane is in direct contact with the ECF and has Na^+^-dependent transport systems and a Na^+^ gradient that can move metabolites out of the ECF against a concentration gradient. The luminal membrane has, primarily, facilitative transport systems that allow molecules to enter and exit the endothelial cells.
